# Blood pressure variability and plasma Alzheimer’s disease biomarkers in older adults

**DOI:** 10.1038/s41598-022-20627-4

**Published:** 2022-10-13

**Authors:** Isabel J. Sible, Belinda Yew, Jung Yun Jang, John Paul M. Alitin, Yanrong Li, Aimée Gaubert, Amy Nguyen, Shubir Dutt, Anna E. Blanken, Jean K. Ho, Anisa J. Marshall, Arunima Kapoor, Fatemah Shenasa, Kathleen E. Rodgers, Virginia E. Sturm, Elizabeth Head, Alessandra Martini, Daniel A. Nation

**Affiliations:** 1 Department of Psychology, University of Southern California, Los Angeles, CA 90089 USA; 2 Institute for Memory Impairments and Neurological Disorders, University of California, Irvine, 4201 Social and Behavioral Sciences Gateway, Irvine, CA 92697-7085 USA; 3 Davis School of Gerontology, University of Southern California, Los Angeles, CA 90089 USA; 4 San Francisco Veterans Affairs Health Care System, San Francisco, CA 94121 USA; 5 Department of Psychiatry, University of California, San Francisco, San Francisco, CA 94158 USA; 6 Department of Psychological Science, University of California, Irvine, Irvine, CA 92697 USA; 7 Department of Pharmacology, Center for Innovation in Brain Science, The University of Arizona, Tucson, AZ 85721 USA; 8 Department of Neurology, University of California, San Francisco, San Francisco, CA 94158 USA; 9 Global Brain Health Institute, University of California, San Francisco, San Francisco, CA 94158 USA; 10 Department of Pathology & Laboratory Medicine, University of California, Irvine, Irvine, CA 92697 USA

**Keywords:** Biomarkers, Ageing

## Abstract

Blood pressure variability is an emerging risk factor for Alzheimer’s disease in older adults, independent of average blood pressure levels. Growing evidence suggests increased blood pressure variability is linked to Alzheimer’s disease pathophysiology indexed by cerebrospinal fluid and positron emission tomography markers, but relationships with plasma Alzheimer’s disease markers have not been investigated. In this cross-sectional study of 54 community-dwelling older adults (aged 55–88, mean age 69.9 [8.2 SD]), elevated blood pressure variability over 5 min was associated with lower levels of plasma Aβ_1–42_ (standardized ß =  − 0.36 [95% CI − 0.61, − 0.12]; *p* = 0.005; adjusted *R*^2^ = 0.28) and Aβ_1–42_: Aβ_1–40_ ratio (ß =  − 0.49 [95% CI − 0.71, − 0.22]; *p* < 0.001; adjusted *R*^2^ = 0.28), and higher levels of total tau (ß = 0.27 [95% CI 0.01, 0.54]; *p* = 0.04; adjusted *R*^2^ = 0.19) and Ptau_181_:Aβ_1–42_ ratio (ß = 0.26 [95% CI 0.02, 0.51]; *p* = 0.04; adjusted *R*^2^ = 0.22). Findings suggest higher blood pressure variability is linked to plasma biomarkers of increased Alzheimer’s disease pathophysiology.

## Introduction

Both high and low blood pressure (BP) are associated with Alzheimer’s disease (AD) dementia risk and pathology^1,2^, and even small improvements in BP control may have the potential to minimize deleterious health outcomes on a world-wide scale^3,4^. Beyond modifying average BP levels, there has been recent interest in BP variability (BPV) as a risk factor for dementia, in part due to BP’s highly variable nature and its potential therapeutic implications^5–8^. Emerging evidence suggests elevated BPV over the longer term (e.g., over months to years or “visit-to-visit” BPV) and shorter term (e.g., over minutes, days) is associated with cognitive impairment and decline^8^, incidence and progression of dementia, including AD and vascular dementia^7,9–12^, cerebrovascular disease^13–15^, stroke^16,17^, and AD pathology^18^, independent of average BP levels^8^. Further evidence indicates these relationships may be especially pronounced in individuals at increased genetic risk for AD through the apolipoprotein (APOE) e4 allele^19–21^.

Amyloid-beta (Aβ), phosphorylated tau (Ptau), and total tau are well-studied indicators and predictors of AD-related neurodegeneration, even in individuals without cognitive impairment^22^, and are already being included as endpoints in clinical trials that increasingly focus on preclinical or early stages of AD^23^. There are several methods available to measure these hallmark AD biomarkers: cerebrospinal fluid (CSF), positron emission tomography (PET), post-mortem evaluation, and most recently, plasma from blood samples. Growing evidence suggests elevated BPV is associated with CSF^21^, PET^20^, and postmortem markers^15,18^ of AD pathology. However, relationships with the newer plasma markers are unknown and could represent a less invasive and lower cost method to characterize AD biomarkers. Additionally, the majority of studies on BPV and markers of AD have used visit-to-visit measures of BPV^15,18,20,21^, and relationships with continuous, acute BPV are understudied. The present study examined links between short-term BPV (continuously collected over a 5-min resting period) and plasma AD biomarkers in a sample of community-dwelling, independently living older adults.

## Results

A total of 54 older adults were included in the present study. Clinical and demographic information about the sample is summarized in Table 1.

**Table 1:** Demographic and clinical information.

	Total sample (*N* = 54)	Low BPV* (*n* = 28)	High BPV* (*n* = 26)
Age (years)	69.9 (8.2)	68.4 (7.7)	71.6 (8.5)
Sex (male/female)	20/33	11/17	9/16
**Race/ethnicity (*n*, %)**			
White	40 (74.1%)	20 (71.4%)	20 (76.9%)
Black	4 (7.4%)	3 (10.7%)	1 (3.9%)
Asian	7 (13.0%)	2 (7.1%)	5 (19.2%)
Other	3 (5.6%)	3 (10.7%)	0 (0.0%)
Education (years)	16.4 (2.5)	16.2 (2.7)	16.7 (2.3)
APOE-ϵ4 carriers (*n*, %)	24 (44.4%)	14 (50.0%)	10 (38.5%)
DRS-2 total (scaled score)	11.8 (2.2)	11.7 (2.4)	12.0 (2.0)
Body mass index (kg/m^2^)	25.9 (5.2)	25.9 (5.3)	25.9 (5.2)
**Fazekas score (*n*, %)**			
0	4 (7.6%)	2 (7.1%)	2 (7.7%)
1	28 (52.8%)	13 (46.4%)	15 (57.7%)
2	17 (32.1%)	12 (42.9%)	5 (19.2%)
3	4 (7.6%)	1 (3.6%)	3 (11.5%)
Antihypertensive use (*n*, %)	18 (33.3%)	7 (25.0%)	11 (42.3%)
Systolic BP (mmHg)	131.0 (16.9)	130.8 (16.6)	131.2 (17.6)
Diastolic BP (mmHg)	74.0 (13.5)	74.2 (18.3)	75.8 (12.7)
Systolic BPV (mmHg)	9.3 (0.9)	8.5 (0.5)	10.0 (0.6)
Diastolic BPV (mmHg)	7.4 (0.5)	7.0 (0.4)	7.8 (0.3)
Aβ_1–42_ (pg/mL)	9.7 (3.6)	10.5 (3.0)	8.7 (3.9)
Ptau_181_ (pg/mL)	2.9 (1.5)	2.9 (1.7)	2.9 (1.4)
Total tau (pg/mL)	2.9 (1.0)	2.6 (0.9)	3.2 (1.0)
Ptau_181_: Aβ_1–42_	0.3 (0.2)	0.3 (0.2)	0.4 (0.2)
Aβ_1–42_: Aβ_1–40_	0.05 (0.02)	0.06 (0.02)	0.04 (0.02)

Means and SDs shown unless otherwise indicated.

*APOE e4* apolipoprotein e4, *DRS-2* Dementia Rating Scale-second edition, *BP* blood pressure, *BPV* blood pressure variability, *Ptau*_*181*_ phosphorylated tau.

*Low/high BPV calculated as below/above the mean BPV from the overall sample.

**Figure 1:**
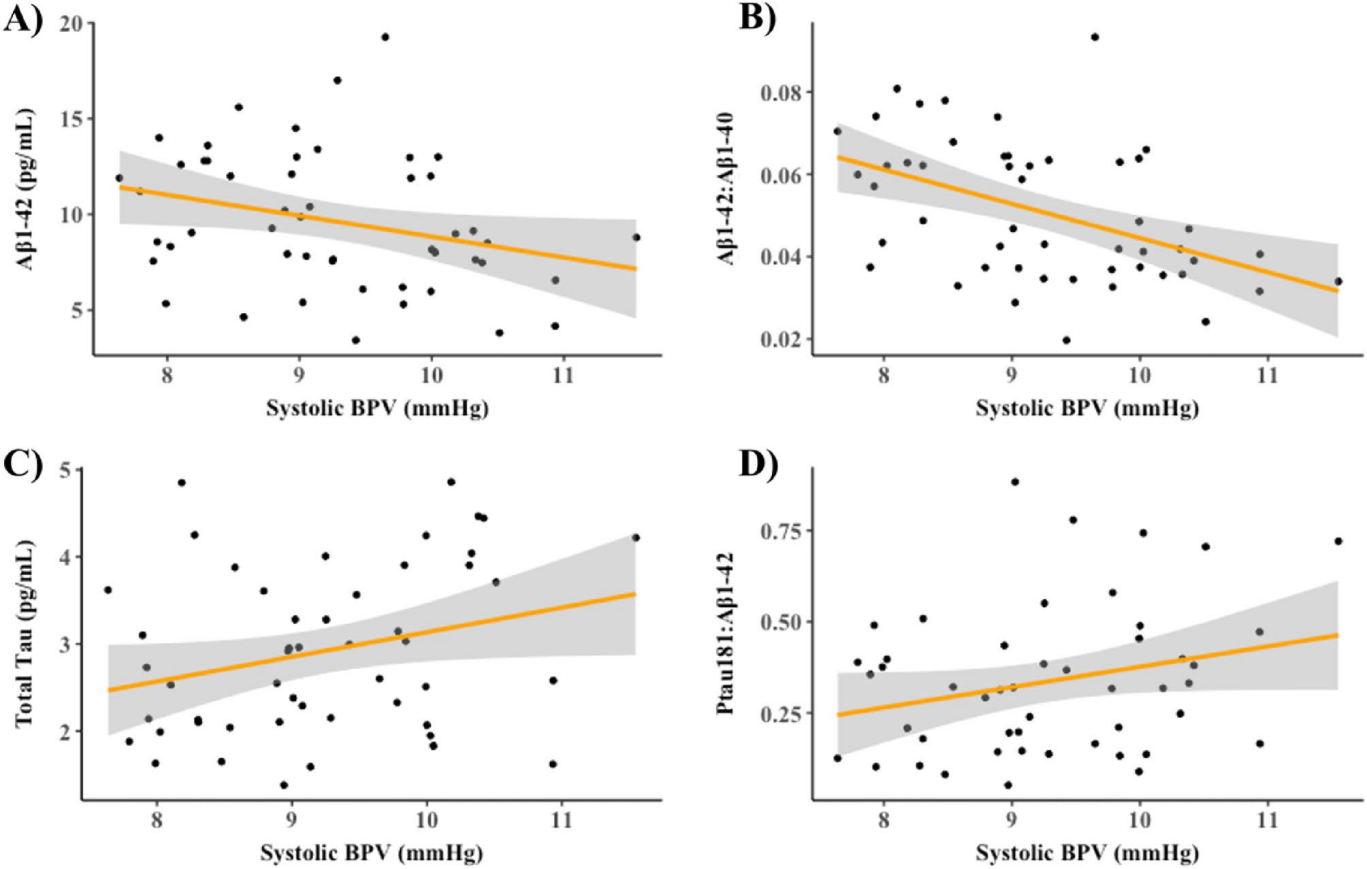
Elevated short-term systolic BPV is associated with plasma AD biomarkers in older adults. Scatterplots display the relationship between short-term systolic BPV and (**A**) Aβ_1–42_, (**B**) Aβ_1–42_: Aβ_1–40_, (**C**) total tau and (**D**) Ptau_181_: Aβ_1–42_ plasma AD biomarker levels in older adults. Lines are shaded with 95% CI. Plasma values were adjusted for age, sex, and APOE e4 carrier status. *BPV* blood pressure variability.

Elevated systolic BPV was significantly associated with lower levels of Aβ_1–42_ (standardized beta (ß) =  − 0.36 [95% CI − 0.61, − 0.12]; *p* = 0.005; adjusted *R*^2^ = 0.28) (Fig. 1A), lower ratio of Aβ_1–42_:Aβ_1–40_ (ß =  − 0.49 [95% CI − 0.71, − 0.22]; *p* < 0.001; adjusted *R*^2^ = 0.28) (Fig. 1B), higher levels of total tau (ß = 0.27 [95% CI 0.01, 0.54]; *p* = 0.04; adjusted *R*^2^ = 0.19) (Fig. 1C), and higher ratio of Ptau_181_:Aβ_1–42_ (ß = 0.26 [95% CI 0.02, 0.51]; *p* = 0.04; adjusted *R*^2^ = 0.22) (Fig. 1D), after controlling for age, sex, and APOE e4 carrier status. BPV was not significantly associated with levels of Ptau_181_ (ß =  − 0.009 [95% CI − 0.29, 0.27]; *p* = 0.95; adjusted *R*^2^ = 0.11) (data not shown). Findings with diastolic BPV were similar (see Supplementary Table 1).

### Sensitivity analyses

The majority of findings remained significant when controlling for (1) cerebrovascular disease by Fazekas score/white matter hyperintensity severity, (2) global cognitive ability by DRS-2 scaled total score, (3) years of education, (4) body mass index (BMI), and (5) antihypertensive medication use (see Supplementary Table 2). Findings remained significant after FDR correction for Aβ_1–42_ (*p* = 0.01) and Aβ_1–42_:Aβ_1–40_ ratio (*p* = 0.002), and were no longer significant for total tau (*p* = 0.05) or Ptau_181_:Aβ_1–42_ ratio (*p* = 0.05).

## Discussion

Findings suggest elevated BPV over a period of a few minutes is associated with plasma AD biomarker levels in a sample of community-dwelling older adults, independent of average BP levels. Results are consistent with growing evidence that BPV may be a useful marker of vascular dysfunction related to AD^5–10,15,19–21,24–28^. Findings add to previous studies on BPV using CSF^21^ and PET^20^ AD biomarkers.

Mechanisms underlying BPV remain unclear and fluctuations in BP are common and complex^29^. BP levels change in the face of environmental, physical, and emotional factors and have an inverse relationship with heart rate variability^30^, a well-studied marker of cardiovascular and emotional health^31^. Just as age-related declines in heart-rate variability are thought to reflect diminished vagal tone and autonomic control^31,32^, BP becomes increasingly variable over time likely due to a combination of physiological factors such as baroreceptor reflex sensitivity and arterial stiffness^16,29,30,33–37^. Arteries tend to stiffen with age as a result of chronic mechanical stress on arterial walls exerted with each heartbeat^38^. Arterial stiffening may alter pulse wave dynamics, potentially causing a buildup or otherwise erratic flow of blood and tissue perfusion^5,36,39^. Disrupted blood flow may be particularly detrimental to organs with high metabolic demand like the brain^35,36,38,40–44^. The brain’s smaller cerebral arterioles and capillaries may be the most vulnerable, where the majority of nutrient transfer, nutrient influx, and waste clearance takes place across the blood–brain barrier, which could be related to the present study findings. Additionally, disrupted blood flow could be a harbinger of microvascular damage and a large body of evidence suggests BPV is associated with cerebrovascular disease burden on neuroimaging^13,14,45^ and postmortem evaluation^15,18^.

Other evidence indicates BPV may be related to endothelial dysfunction^27,46^, which could underlie the findings with Ptau in the current study. Specifically, a recent rodent study found that loss of nitic oxide due to high dietary salt intake leads to changes in neuronal enzymatic function that promote tau phosphorylation^47^. Therefore, it is possible that BPV in the present study is associated with Ptau via endothelial nitic oxide deficiency, which is in turn strongly linked with hypertension^47^.

Prior work on BPV and AD pathophysiology using CSF^21^ and PET^20^ found weaker, if any, links with Aβ when compared to findings with Ptau. In contrast, BPV was strongly associated with plasma markers of Aβ_1–42_ and Aβ_1–42_:Aβ_1–40_. It remains unclear whether the discrepancy between BPV relationships with central (e.g., CSF, PET) versus peripheral (plasma) Aβ markers may be related to differences in metabolic factors impacting central versus peripheral Aβ levels. Interestingly, Aβ is cleared in both the brain and in the kidneys, and damage to the kidneys is independently related to elevated BPV^48^. Additionally, chronic kidney disease may impact clearance of proteins and has been associated with elevated levels of plasma Aβ_1–42_ and Aβ_1–40_ as well as plasma Ptau_181_ and Ptau_217_^49^. Therefore, it is possible that plasma/peripheral Aβ may be more impacted by kidney function than central nervous system measures of Aβ, although this remains an open question for future investigation.

It is also true that prior CSF and PET studies used BPV measured over a period of months (e.g., “visit-to-visit” BPV) and the present investigation is the first to examine links between short-term BPV over a period of a few minutes and plasma markers of AD. Thus, it is possible that acute BPV reflects different physiological mechanisms than visit-to-visit measures. For example, short-term BPV is thought to reflect sympathetic nervous system overactivation and peripheral noradrenaline signaling to a greater degree than visit-to-visit measures, which may be of relevance to the relationship between BPV and Aβ levels^29,37,48^.

Alternatively, neurodegeneration of autonomic centers in the brain could be related to both BP fluctuations and AD pathophysiology^28^. The cross-sectional nature of the present study limits our ability to assess directionality or potential mechanisms and future studies are needed to elucidate ways in which BPV may be linked with increased dementia risk. Nevertheless, study findings suggest BPV may be related to plasma AD biomarkers in older adults without major neurocognitive disorder, with potential therapeutic implications. A few studies indicate differential class effects of BPV on risk for stroke^50,51^, independent of traditionally studied average BP levels, but more research is needed. Antihypertensive treatment decisions that consider the variability in BP levels could improve precision-medicine approaches to dementia care^52,53^.

A strength of the present study is the focus on blood-based AD biomarkers, a promising alternative to more invasive and costly markers of AD pathophysiology (i.e., CSF, PET) that could make their way into a wider range of clinical settings. Relatedly, BPV, whether assessed continuously over a few minutes, via ambulatory monitoring, or at routine clinical visits, is a readily available index of vascular health^29^ with growing links to dementia risk^8,11,54^. The present study used acute, continuous BPV and adds to the literature using BPV measured over longer periods. Findings were present even in this sample of community-dwelling older adults, consistent with prior reports that elevated BPV may occur before the onset of major neurocognitive dysfunction^24^. However, investigating relationships in study samples with more advanced disease may be helpful. There are several limitations worth noting. First, the sample size is relatively small and additional studies with larger sample sizes are needed. Relatedly, it was not possible to assess for potential antihypertensive medication class effects. Second, BP measurements and plasma samples were collected at two sites, USC and UCI, which could introduce measurement error. Additionally, BP fluctuations occur for many reasons, some of which we were not able to control for in the present study (e.g., stimulant intake, medication use, pain, perceived stress). BPV was calculated over a period of a few minutes, consistent with most prior studies on short-term BPV^29^. However, the study of BPV is an emerging field and there is no current gold standard of time to measure short-term BPV. Finally, the study sample was generally representative of community-dwelling older adults in the local region in terms of cerebrovascular disease^55^ (e.g., 59.3% had Fazekas scores ≤ 1 suggesting minimal cerebrovascular disease burden, 38.9% had Fazekas scores ≥ 2 suggesting moderate to severe cerebrovascular disease burden), education (mean 16.4 SD 2.5 years [range 9–20 years]), BMI (mean 25.9 SD 5.2), and race/ethnicity (74.1% non-Hispanic White). However, replicating these findings in larger and more heterogeneous cohorts is an important area for future research.

## Conclusions

Elevated short-term BPV, independent of average BP levels, in a sample of community-dwelling older adults is related to blood-based markers of Aβ and tau-mediated neurodegeneration. BPV may be a readily accessible but understudied vascular factor associated with AD pathophysiology.

## Methods

### Participants

Study participants were drawn from the Vascular Senescence and Cognition Lab at the University of Southern California (USC) and University of California Irvine (UCI), an ongoing research study (n = 126) of vascular contributions to cognitive decline and dementia. Participants were recruited from the community via flyers and related research list-serves at USC and UCI. Inclusion criteria included aged 55–90 and living independently in the greater Los Angeles and Orange County areas. Participants were excluded for history of dementia, stroke, traumatic brain injury, learning disability, or other systemic or neurological disorder known to affect the central nervous system. Additionally, all research participants underwent neuropsychological testing that included the Mattis Dementia Rating Scale-2 (DRS-2)^56^, a widely used measure of global cognition. Remaining eligible participants were further excluded based on a DRS-2 total score ≤ 126, an established cutoff to rule out major neurocognitive impairment^56^. The study was approved by the Institutional Review Board at USC and UCI and all participants provided their written informed consent. All methods were performed in accordance with the relevant guidelines and regulations.

Of the total 126 participants enrolled in the ongoing study, 70 did not have valid BPV data and 2 did not have plasma AD biomarker data available. Therefore, 54 older adult participants (aged 55–88) who underwent continuous BP monitoring over a 5-min resting period and venipuncture to determine levels of plasma AD biomarkers were included in the present study.

### Measures

#### BP assessment

BP was collected continuously using a Biopac® BP monitoring device during a 5-min resting period in which participants were in the supine position. Data were processed offline using a custom pipeline scripted in AcqKnowledge® that excluded outliers and instances of noise (e.g., signal dropout due to sensor interference), as described elsewhere^25^. Intraindividual BPV was calculated as variation independent of mean (VIM), a commonly used measure of BPV uncorrelated with mean BP levels^7,24,26,57–59^. In our study, BPV was not significantly correlated with average BP levels (systolic: *r* = 0.12, *p* = 0.40; diastolic: *r* = 0.28, *p* = 0.06). VIM was calculated as: VIM = standard deviation (SD)/mean^*x*^, where the power *x* was derived from non-linear curve fitting of BP SD against average BP using the nls package in R Project, as previously described^15,24,26,57^.

#### Plasma AD biomarkers assessment

Participants underwent venipuncture after an overnight 12-h fast, and within 1–2 h before BP monitoring. Blood samples were collected in EDTA tubes and used to determine levels of plasma AD biomarkers Aβ_1–42_, Aβ_1–40_, Ptau_181_, and total tau. Ptau_181_ samples were processed using the assay Simoa® pTau-181 Advantage V2 Kit (Quanterix). Accepted ranges for Ptau^181^ values were 0–424 pg/mL. Aβ_1–42_, Aβ_1–40_, and total tau samples were processed using the assay Simoa® Neurology 3-Plex A Advantage Kit (Quanterix). Accepted ranges were as follows: Aβ_1–42_ = 0–240 pg/mL, Aβ_1–40_ = 0–560 pg/mL, total tau = 0–400 pg/mL. Ratios were determined for Ptau_181_:Aβ_1–42_ and Aβ_1–42_:Aβ_1–40_.

#### White matter hyperintensity assessment

Nearly all participants (n = 53 out of 54) also underwent T2-fluid attenuated inversion recovery (FLAIR) MRI sequence for the evaluation of white matter lesions, as previously described^25^. Briefly, the following imaging parameters were used: TR = 10,000 ms; TE = 91 ms; TI = 2500 ms; slice thickness = 5.0 mm; flip angle = 150°; field of view = 220 mm. Fazekas scores^60^ were visually determined by the same rater, blinded to all other study measures, and used to calculate severity of white matter lesions.

#### Other measurements

Blood samples from venipuncture were also used to determine APOE e4 carrier status (≥ 1 e4 allele), as previously prescribed^61^. Genomic DNA was extracted using the PureLink Genomic DNA Mini Kit (Thermo). The isolated DNA concentration was determined using a NanoDrop One (Thermo). DNA was then stored at − 80 °C for long-term storage. Isolated DNA was first diluted to a concentration of 10 mg/μL. PCR reactions were performed in a final volume of 25 μL containing 25 ng DNA, 0.5 μM of both forward and reverse primers (forward: ACGGCTGTCCAAGGAGCTG; reverse: CCCCGGCCTGGTACACTG), and 1× SYBR Green Master Mix (Qiagen) diluted in H2O. For the amplification, a T100 Thermal Cycler (BioRad) was used with the following settings: 95 °C for 10 min; 32 cycles of 94 °C for 20 s, 64 °C for 20 s, and 72 °C for 40 s; followed by 72 °C for 3 min. 15 μL of the DNA PCR product was digested with Hhal-fast enzyme at 37 °C for 15 min. The digested PRC product was added to a 3% agarose gel in 1× borax buffer for gel electrophoresis. The gel was run at 175 V for 25 min and visualized on ChemiDoc (BioRad) with a GelRed 10,000× gel dye. Height (m) and weight (kg) were determined from study screening and used to calculate body mass index (BMI [kg/m^2^]). Study screening also determined self-reported antihypertensive use and participants were categorized as those taking antihypertensive medication (all classes) vs those who were not. Total DRS-2^62^ raw scores (max score = 144) were converted to age-corrected scaled scores.

### Statistical analysis

BPV and plasma AD biomarker outliers that were ± 3 SD from the mean were removed. One participant was excluded (Ptau_181_:Aβ_1–42_ was + 3 SD from the mean). Multiple linear regression was used to investigate relationships between BPV and individual plasma AD biomarkers (and their ratios). All models included the potentially confounding variables age, sex, and APOE e4 carrier status. Sensitivity analyses tested the robustness of findings by controlling for (1) Fazekas score, (2) DRS-2 scaled total score, (3) years of education, (4) BMI, and (5) antihypertensive medication use (see Supplementary Materials). These are commonly used covariates in other BPV studies^8,14,25^ and were entered one at a time into regression models. Systolic BPV findings are reported in the main text and diastolic findings are reported in Supplementary Materials. Multiple comparison corrections using the False Discovery Rate (FDR) method for significant findings was set at *p* < 0.05. All analyses were 2-sided with significance set at *p* < 0.05 and were carried out in R Project^63^.

## Supplementary Information

Supplementary Tables.

## Data Availability

Data are available by request to Daniel Nation.
