# Supplementary material for: Blood pressure variability and plasma Alzheimer’s disease biomarkers in older adults
Source: Sci Rep. 2022 Oct 13;12:17197. doi: 10.1038/s41598-022-20627-4 (PMC9561652; doi:10.1038/s41598-022-20627-4)
Supplement: Supplementary file 1 — Supplementary Tables. [file 41598_2022_20627_MOESM1_ESM.docx]

SUPPLEMENTARY MATERIALS

**Supplementary Table 1.**

Diastolic BPV analyses

|  | **ß (95% CI)** |
| --- | --- |
| Aβ_1-42_ (pg/mL) | **-.35 (-.60, -.09)** |
| Total tau (pg/mL) | **.36 (.09, .65)** |
| Ptau_181_:Aβ_1-42_ | .20 (-.06, .46) |
| Aβ_1-42_:Aβ_1-40_ | **-.46 (-.72, -.21)** |

ß and 95% confidence interval for findings with diastolic BPV. Bolded items indicate significant findings.

Abbreviations: Ptau = phosphorylated tau

**Supplementary Table 2.**

Sensitivity analyses of systolic BPV findings.

| **Added covariate** | **Aβ_1-42_ (pg/mL)** | **Total tau (pg/mL)** | **Ptau_181_:Aβ_1-42_** | **Aβ_1-42_:Aβ_1-40_** |
| --- | --- | --- | --- | --- |
| Cerebrovascular disease severity | **ß = -.35 [95% CI -.61, -.09]** | **ß = .28 [95% CI .004, .55]** | ß = .21 [95% CI -.04, .46] | **ß = -.45 [95% CI -.71, -.20]** |
| DRS-2 score | **ß = -.36 [95% CI -.61, -.10]** | **ß = .32 [95% CI .07, .57]** | ß = .25 [95% CI -.01, .51] | **ß = -.49 [95% CI -.75, -.23]** |
| Education | **ß = -.37 [95% CI -.62, -.12]** | **ß = .30 [95% CI .04, .55]** | **ß = .27 [95% CI .02, .52]** | **ß = -.47 [95% CI -.72, -.22]** |
| BMI | **ß = -.35 [95% CI -.60, -.09]** | ß = .22 [95% CI -.02, .47] | **ß = .26 [95% CI .01, .52]** | **ß = -.45 [95% CI -.70, -.20]** |
| Antihypertensive use | **ß = -.37 [95% CI -.66, -.08]** | ß = .24 [95% CI -.07, .54] | ß = .26 [95% CI -.02, .53] | **ß = -.46 [95% CI -.75, -.18]** |

ß and 95% confidence interval for findings with systolic BPV. Bolded items indicate significant findings.

Abbreviations: DRS – 2 = Dementia Rating Scale – 2^nd^ edition; BMI = body mass index; Ptau = phosphorylated tau
